# CFRP Fatigue Damage Detection by Thermal Methods

**DOI:** 10.3390/ma15113787

**Published:** 2022-05-26

**Authors:** Marta De Giorgi, Riccardo Nobile, Fania Palano

**Affiliations:** 1 Department of Engineering for Innovation, University of Salento, 73100 Lecce, Italy; riccardo.nobile@unisalento.it; 2 ENEA, Centro Ricerche di Brindisi c/o Cittadella della Ricerca S.S.7 Appia km 706, 72100 Brindisi, Italy; fania.palano@enea.it

**Keywords:** composites, fatigue, thermal methods, damage

## Abstract

In this work, the fatigue damage of CFRP uniaxial composite specimens were studied using thermal methods to determine the fatigue behavior. The aim was to evaluate the fatigue damage as a function of the number of cycles. Consequently, the damage process was studied in terms of a global indicator, considering the stiffness decay, and in terms of local parameters, considering the evolution of temperature maps acquired during the fatigue tests. A direct correlation between the damage index, corresponding to 90% of the fatigue life, and the temperature variation of the most stressed area was found. Another parameter taken into consideration was the heating rate during the application of the first thousands cycles. This parameter was proportional to the stress amplitude, making it a useful parameter since it refers to the initial part of the specimen fatigue life.

## 1. Introduction

Fiber-based composites, a relatively new type of material, are proving particularly interesting to the aerospace and automotive sectors due to their ability to decrease component weight, increase performance, and improve impact tolerance/crashworthiness, all factors that are important in lowering fuel emissions and increasing occupant safety. More recently, increasingly sectoral and promising applications have emerged in the field of civil engineering, such as in bridge structures and the oil extraction industry [1,2]. In almost all application fields, components and structures are subject to fatigue phenomena. Over the past few decades, the fatigue performances of CFRP and GFRP have been investigated [3,4,5,6], showing that CFRP has excellent fatigue performance compared to GFRP. On the other hand, the fatigue life of FRP is highly dependent on the properties of the material system such as fiber–resin matrix performance, lay-up sequence, and residual stress from the manufacturing process [7,8,9]. All these factors have relevant influence on fatigue crack initiation and propagation. The fatigue failure mechanism of FRP is complex and fatigue damage includes fiber breakage, resin matrix cracking, fiber–resin debonding, and delamination [10]. Furthermore, stress level has a significant effect on the fatigue damage mechanism and fatigue life. Therefore, the fatigue behavior of a polymer matrix composite material is the result of a set of alterations, depending on the load and the number of cycles applied, which occur in the material both globally and locally and which are responsible for the damage. Often, the area affected by damage is only a small portion of the entire volume of the component.However it can lead to a sudden break. In such circumstances, a “global” measure of the damage may not provide any reliable estimate of the extent of the evolving phenomena. During the application of fatigue loads, the material undergoes progressive damage which often does not manifest itself with significant deformations but is linked to localized and sometimes sudden dissipation phenomena. The areas where the damage accumulates are always affected by dissipative phenomena that can be highlighted by starting from temperature maps. The development of damage in a composite laminate can be schematized in three phases [11]. In the first phase, there is the primary fracture mechanism, whereby the matrix breaks along the fibers which are oriented in a different direction with respect to the load. The number of cracks increases exponentially with the load and tends to reach a saturation level which is a characteristic of the laminate, called the “Characteristic Damage State” (CDS). Its achievement indicates the end of the primary fracture phase. Subsequently, transverse fractures to the primary ones occur. These secondary fractures cause the onset of the interlaminar fracture, initially in limited areas and later more widely. The subsequent development of the damage is instead strongly localized, has an unstable growth, involves the breaking of the fibers arranged in the direction of the load, and leads to the collapse of the laminate. For both the pre-CDS and post-CDS damage phases, experimental models have been developed that help to evaluate the evolution of the damage and the residual life of the laminate itself. Damage mechanisms cause degradation of the composite material’s elastic properties without causing sudden failure. In a multidirectional laminate made of unidirectional plies, the principal causes of stiffness degradation are crack initiation and propagation in the matrix’s off-axis layers [12,13,14,15,16,17,18].

Composite fatigue behavior can therefore be related to stiffness and strength degradations resulting from damage accumulation. Several empirical evolution models have been developed that describe a gradual macroscopic reduction in the laminate stiffness related to the damage evolution on a microscopic scale [19,20]. Arafat et al. [21] implemented Shokrieh’s [22] empirical stiffness and strength degradation model as the user material subroutine UMAT in the ABAQUS FE software, validating the procedure with test data from the literature. The authors conclude that Shokrieh’s model provides a good fatigue life estimation for preliminary designs. Carraro and Quaresimin [23] studied stiffness loss in a generic cracked symmetric laminate, proposing an analytical model for the elastic properties evaluation of a multidirectional symmetric laminate with off-axis cracks. Stiffness decrease estimations were in very good agreement with experimental data. In [24], the authors presented a damage-based design procedure to predict damage evolution and the stiffness degradation in polymeric composite laminates under fatigue loading. The proposed models evaluated the lifetime associated with the principal damage mechanisms (off-axis cracks, delamination, and fiber failure). Moreover, the stiffness degradation caused by fatigue damage evolution was accurately explained. In [25], material property degradation was determined by measuring the change in specimen stiffness to analyze the progression of fatigue damage which is correlated to micro- and macroscale damage mechanisms and biaxial fatigue loading parameters. Russo et al. [26] studied the fatigue response of composite materials using numerical methods with Shokrieh and Lessard’s residual strength material property degradation model. A finite element methodology was implemented in the commercial software ANSYS MECHANICAL. In [27], a procedure was proposed for studying the damage of composite materials using thermographic metrics. The thermoelastic temperature signal was used as a parameter to assess the stress–strain redistribution in the material. Numerous models relating residual stiffness to FRP composite fatigue behavior are presented in a comprehensive review by Wang and Zhang [28].

In the present work, the study of the fatigue behavior of uniaxial carbon fiber and epoxy resin composite laminate specimens, made with resin transfer molding (RTM) technology, is presented. The innovative aspect of this study is the use of thermal methods to investigate the damage evolution in terms of stiffness decay. The goal is to evaluate the damage as the number of load cycles increases. Differently from the cited works, the damage process was studied here both in global terms, evaluating the decay of the stiffness of the composite, and in local terms, evaluating the variations of the temperature maps as a function of the number of cycles. For this purpose, fatigue tests were conducted at constant amplitude, with R = 0.1 and a load frequency equal to 10 Hz. The thermographic analysis, associated with the study of stiffness degradation, has the purpose of obtaining some parameters useful to highlight the state of localized damage in a phase preceding the appearance of an evident crack or other rapid degradation phenomena: delamination, detachment, or accentuated deformation.

## 2. Materials and Methods

The experimentation was carried out on a unidirectional composite material in carbon fiber and epoxy resin (carbon–epoxy) obtained with resin transfer molding technology. Principal characteristics of the material are summarized in Table 1. Data reported in Table 1 were obtained through tensile tests in the longitudinal direction. No test was carried out in the transversal direction.

**Figure 1:**
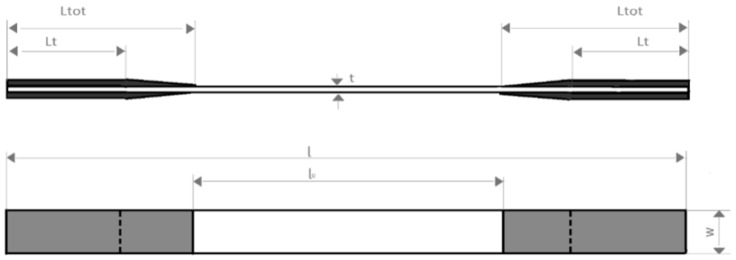
Geometry of the tested specimens.

The specimens tested in this work had tabs at the ends and gauge lengths l_0_ equal to 150 mm, rectangular cross sections with thickness (t) equal to 2 mm, width (w) of 20 mm, and length of the reinforcements (L_t_) equal to 120 mm (Figure 1).

The fatigue characterization of the material was determined by carrying out fatigue tests according to the requirements of the ASTM D 3479 standard [29]. The test parameters were selected on the basis of previous works and on the basis of specific needs. The tests were conducted at constant amplitude, with a stress ratio R = 0.1, using an Instron 8850 servo-hydraulic machine with a load capacity of 250 kN. The test frequency was kept constant for all the specimens and limited to 10 Hz to avoid excessive localized heating near the bonding areas of the reinforcements. Five different load levels were used, briefly identified with the maximum stress value σ_max_ variable between 1100 and 1445 MPa. At least three repeated tests were carried out for each load level. Table 2 reports fatigue test parameters. The maximum number of cycles at which the test was interrupted was 10^7^. Once this condition was reached, each specimen was subjected to a static test in order to evaluate its residual strength.

During the execution of the fatigue tests, the load peaks F_max_, F_min_ and the displacements of the crosshead x_max_, x_min_ were recorded at regular intervals. These data were used to calculate the stiffness of the specimen using the following Formula (1):

(1) $$R=\frac{\Delta F}{\Delta x}$$

where ΔF is the load variation and Δx is the variation in the gauge length l_0_. By doing so, it was assumed that the variation in gauge length could be approximated using the excursion of the crosshead, thus neglecting the deformation of the clamped part and, in general, of the reinforced part.

### Fatigue Tests: Thermographic Analysis

During the application of fatigue loads, the material undergoes progressive damage which often does not manifest itself with significant deformations but is linked to localized and sometimes sudden dissipation phenomena. The areas where the damage accumulates are always affected by dissipative phenomena that can be highlighted by starting from temperature maps. In order to detect these areas, a FLIR 7500 M thermal imaging camera was used, with noise-equivalent thermal sensitivity (NETD) equal to 25 mK and image resolution 320 × 256 pixels, which allowed us to observe the presence and evolution of heterogeneity in the internal part of the material as an indication of localized damage and to follow its propagation until the final break.

Due to the long duration of the tests, especially those at the lowest load levels near the fatigue limit, it was necessary to limit the recording time. It was decided to record the first 20 s of each test in order to be able to capture the thermal transient and to make recordings at regular intervals for a total duration of 1 s at an acquisition frequency of 200 Hz and an integration time of 1000 µs. Taking into account that the frequency of application of the load was equal to 10 Hz, each recording of 1 s corresponded to 10 load cycles, for each of which 20 thermograms were available. The recording interval was set at 5 min for the first 2 h of testing and was thereafter variable between 5 min and 3.5 h depending on the expected duration of the test.

The heat maps obtained showed the onset from the first heating cycles, localized in correspondence with the reinforcement areas; in particular on the one near the lower grip, which is the one connected with the fixed part of the test machine. This heating is due to the slipping phenomena, existing between the reinforcements and the surface of the specimen, caused by the high loads applied.

## 3. Results and Discussions

**Figure 2:**
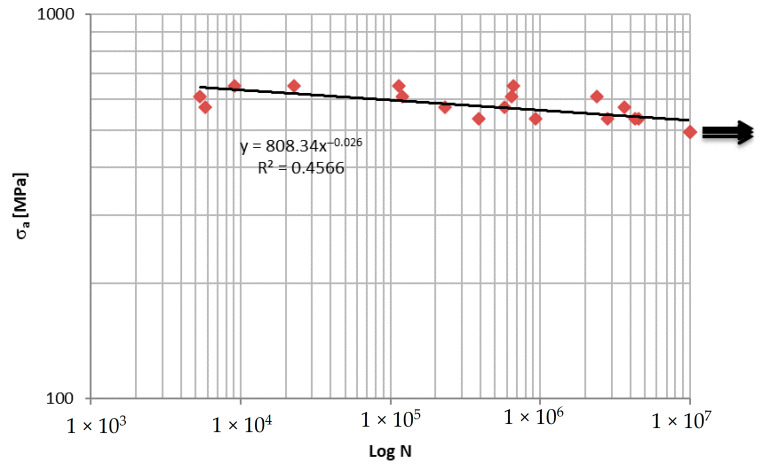
Wöhler curve of the unidirectional composite.

A first evaluation of the fatigue behavior of the tested material was carried out by plotting the Wöhler curve in terms of amplitude stress σ_a_ (Figure 2).

**Figure 3:**
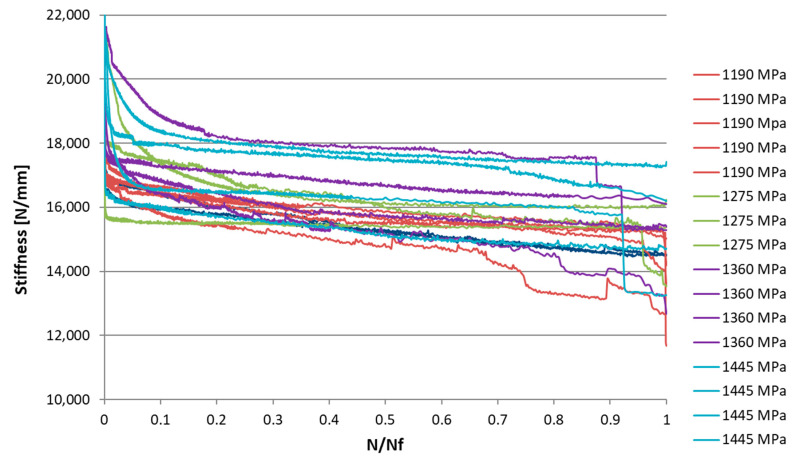
Stiffness trend as a function of the normalized number of cycles.

The diagram in Figure 3 shows the stiffness trend of all tested specimens that came to break.as a function of the normalized number of cycles with respect to the cycles to failure N_f_. 

For all tests, the stiffness showed a more-or-less rapid decrease from the first cycles until it settled with an increase in N. Between 10% and 90% of the useful life, a gradual decrease in stiffness was observed, with an almost linear trend. The slope of this segment was, however, less pronounced than the initial one.

In the final part of the specimen’s life, beyond 90% of the useful life, failure may not be predicted by an increase in the rate of stiffness reduction, but rather by a sudden drop. In many cases the stiffness trend stops abruptly without undergoing a further decrease. This phenomenon is due to the unidirectional structure of the composite and the brittleness of the carbon fibers.

**Figure 4:**
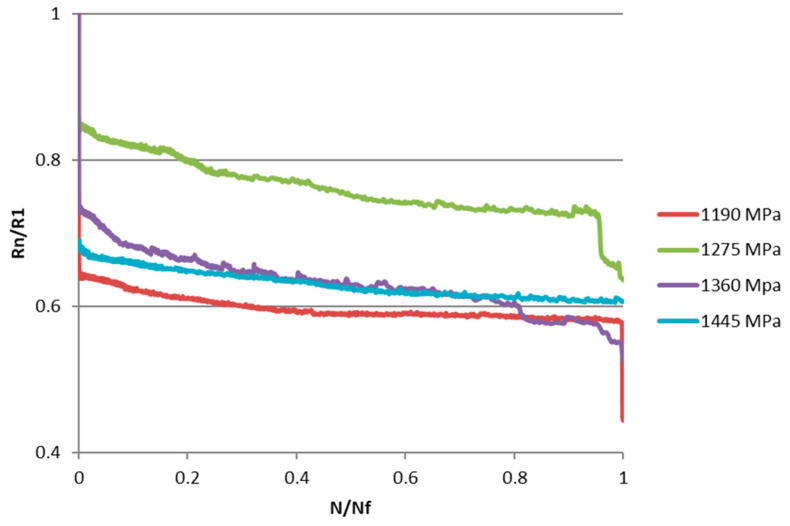
Trend of normalized stiffness for the various load levels applied.

In order to better interpret the results and, in particular, evaluate the effect of the applied load level, the trend of the normalized stiffness was analyzed. Normalized stiffness is defined as the ratio between the stiffness at the n-th cycle and that at the first load cycle R_n_/R_1_. The graph in Figure 4 shows the trend of normalized stiffness as a function of the N/N_f_ ratio for four specimens tested at different load levels. It can be observed that there is no correlation between the reduction in stiffness and the level of load applied.

The evaluation of the progressive damage of the material can be carried out through the following expression which allows for the calculation of the damage D as a function of the variation of the elastic modulus [16,30]:

(2) $$D=\frac{E_{0}-E_{i}}{E_{0}-E_{f}}$$

where E_0_ is the elastic modulus of the material in the initial test conditions; E_i_ is the elastic modulus of the material corresponding to the i-th life cycle; E_f_ is the elastic modulus of the material in the final or failure conditions.

In this work, Equation (2) is rewritten and explained in terms of the variation of stiffness R:

(3) $$D=\frac{R_{0}-R_{i}}{R_{0}-R_{f}}$$

where the same meaning for subscripts is used.

The probabilistic aspect in the study of the composite materials fatigue involves a significant dispersion in the experimental data obtained [31]. As already known, variations in stiffness and damage are closely linked to each other. In the literature [32], it has been proposed that the residual life of a composite material could be estimated based on the evaluation of percentage variation of the stiffness ΔR_%_.

The approach used by the cited method is as follows:

- The linear part of the stiffness trend is identified as a function of the number of cycles;
- The linear trend of the stiffness is traced as a function of the number of cycles through two points: (0; R_0_^lin^) and (0.9 N_failure_; R_90_^lin^); the first point is obtained by extrapolating the trend line up to the ordinate axis, obtaining the ideal stiffness value at cycle 0, and the second point is obtained by considering the stiffness value at 90% of the specimen’s life R_90_^lin^;
- Based on these values, the percentage change in stiffness ΔR_%_ is calculated through the following equation:
  (4) $$\Delta R_{\%}=\frac{R_{0}^{\mathrm{lin}}-R_{90}^{\mathrm{lin}}}{R_{0}^{\mathrm{lin}}}$$

**Figure 5:**
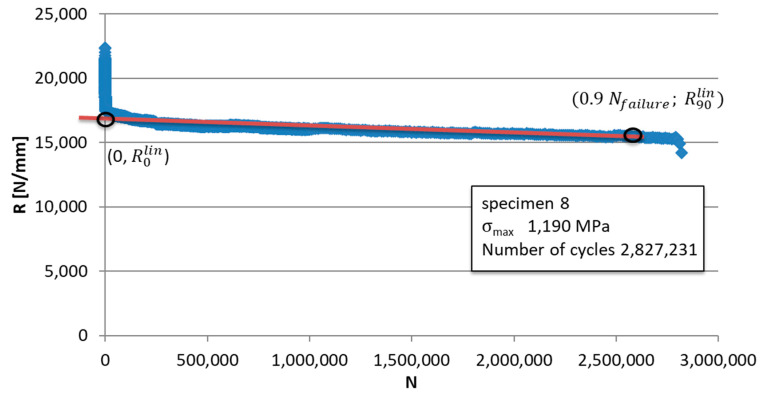
Calculation of the stiffness percentage variation ΔR_%_.

An example of the calculation of the points mentioned above is shown in Figure 5, while the data obtained for all the tested specimens are summarized in Table 3. The specimens that reached run-out were excluded from the calculation of ΔR_%_.

**Figure 6:**
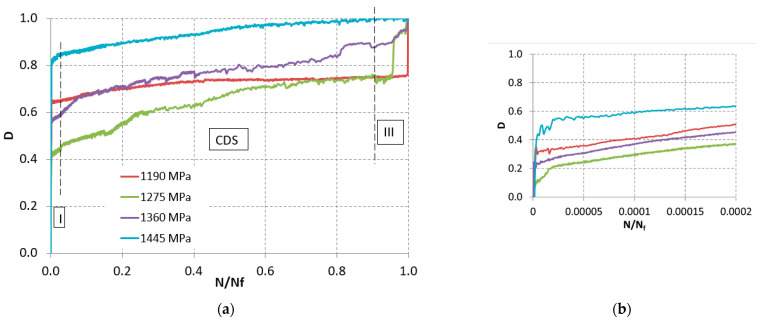
Evolution of the damage index: (**a**) global; (**b**) detail of the first cycles.

From the analysis of the obtained results, it is found that the ΔR_%_ values are affected by high dispersion and, contrary to what was found in other cases, this parameter cannot be used as an indicator of imminent fatigue failure. The ΔR_%_ value associated with failure does not even seem to be related to the level of applied load. Figure 6 reports the evolution of the damage index as a function of the N/N_f_ ratio.

Figure 6 shows that:

- Index D increases proportionally to the applied load. The curves are placed on increasing damage levels as a function of the load, with the exception of the minimum load of 1190 MPa, which is at an intermediate level;
- The three regions corresponding to the different fracture mechanisms in composite laminates are clearly identifiable [16];
- Region I, corresponding to the primary fracture of the matrix, can be highlighted by examining the very first cycles of fatigue (Figure 6b). It is noted that the slope of this phase is very pronounced;
- In region II, called the characteristic state of damage (CDS), the fracture mechanisms between fibers and matrix interact with each other; as the number of cycles increases, the curve presents, in this section, a linear trend but with a less pronounced slope than in region I. It is noted that the behaviors relating to the various load levels present almost identical slopes, generating almost parallel curves;
- Finally, the third region (III) was identified. In it occurs the fracture of the fibers and the consequent rupture of the specimen. This phase takes place in a very short time. There is no warning regarding a possible failure of the specimen in the final part of its useful life;
- The progressive fatigue damage was then followed up by examining the recorded temperature maps. As expected, after a short transient, the temperature of each specimen first undergoes a slow increase, stabilizes at a steady state around a constant value, and then rapidly increases in the terminal phase.

**Figure 7:**
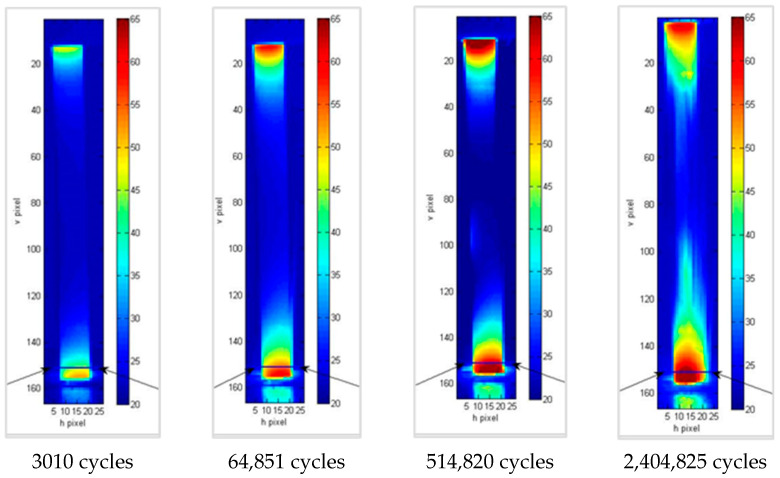
Thermograms at different loading cycles for specimen 1b.

**Figure 8:**
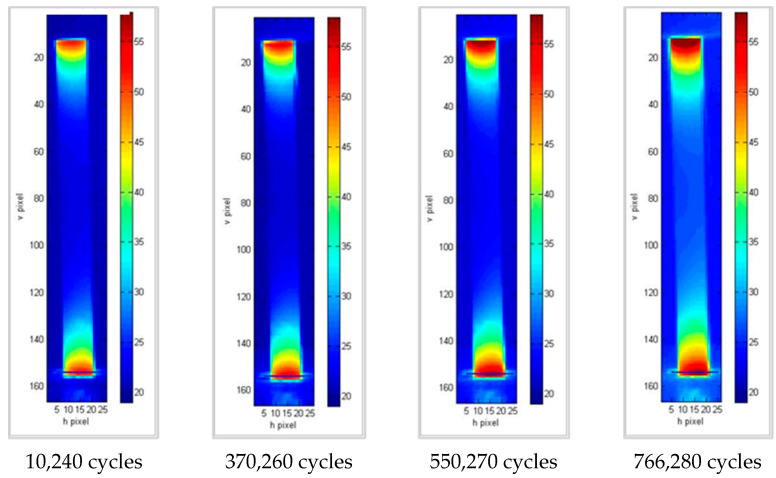
Thermograms at different loading cycles for specimen 14b.

**Figure 9:**
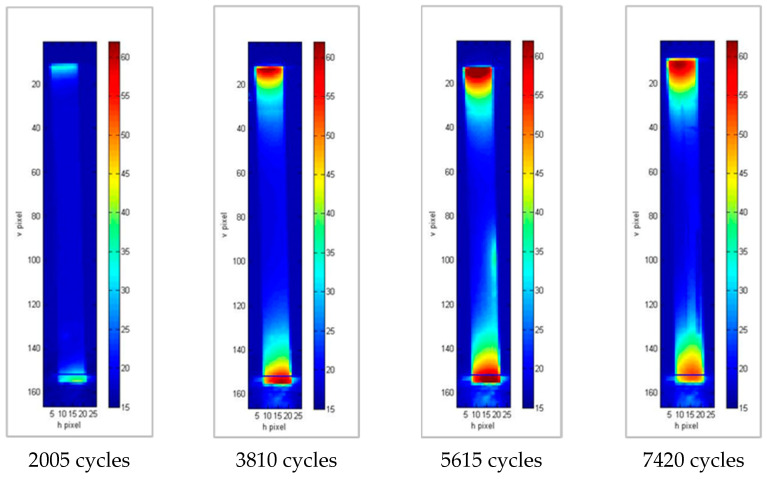
Thermograms at different loading cycles for specimen 8b.

In all cases (Figure 7, Figure 8 and Figure 9), areas with a higher temperature can be identified in correspondence with one of the grips or both. These areas are increasingly extensive and have higher temperatures as the number of cycles increases, highlighting how the application of load cycles produces a local temperature increase which indicates the evolution of damage. The temperature trend was analyzed versus the number of cycles of the higher temperature pixel placed in the lower part of the specimen. This area is the one that undergoes lower displacements, as it is connected to the fixed part of the testing machine. In particular, for each specimen, the increase in temperature compared to that at the initial instant, ΔT, was considered as an index of damage (Table 4).

**Figure 10:**
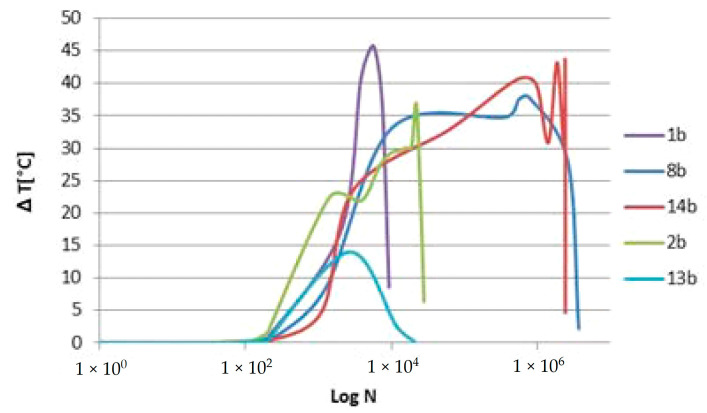
Trend of the temperature increase ΔT as a function of the number of cycles.

Figure 10 shows the temperature trends as a function of the number of cycles for specimens with different endurances. The thermal trends show a limit of the thermal analysis in relation to long-term tests.

**Figure 11:**
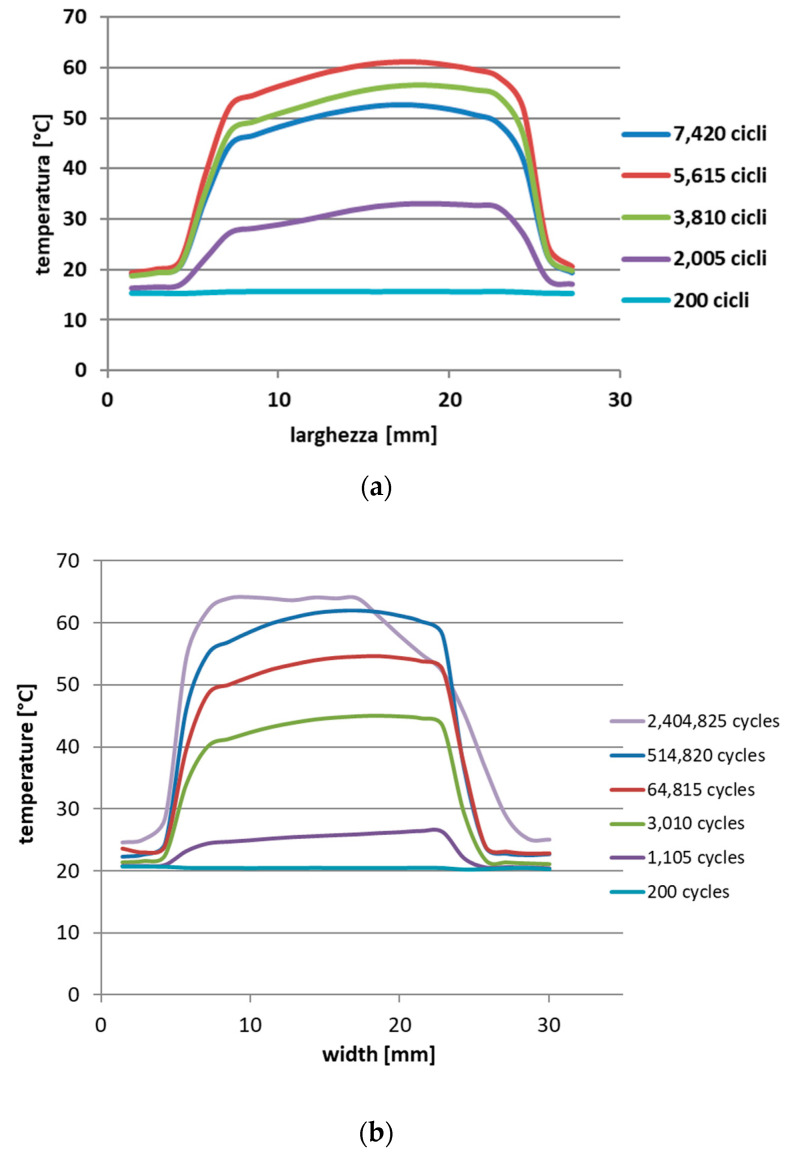
Spatial trend of the thermal profile at different load cycles: (**a**) specimen 1b; (**b**) specimen 14b.

The thermal profile was then observed along a horizontal segment at the selected hot spot to analyze the temperature trend along the width of the specimen and evaluate the increase in the damaged area and the extent of the damage. As an example, the trends for two different load levels are shown (Figure 11), corresponding to very different endurances.

**Figure 12:**
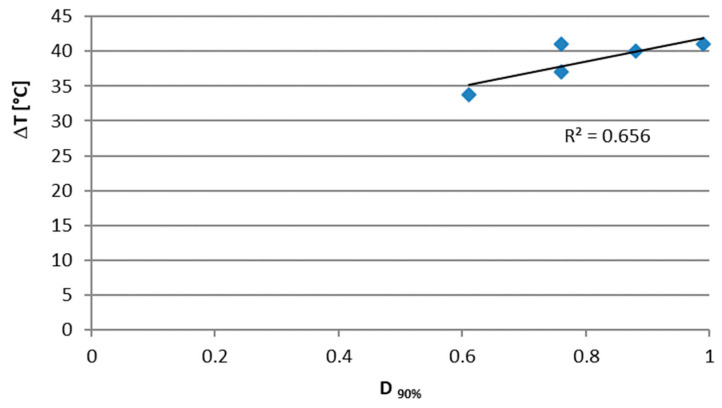
Relationship between the maximum temperature increase and the damage index at 90% of the fatigue life.

By comparing the results obtained from the thermographic analysis to those relating to damage D, a correlation was found between the maximum temperature increase for each specimen and the relative D index at 90% of the fatigue life (Figure 12).

## 4. Conclusions

In the present work, the fatigue behavior of a unidirectional composite material in carbon fiber and epoxy resin made with RTM technology was evaluated. In particular, after obtaining the Wöhler curve, the damage was evaluated in terms of stiffness variation. It was verified that the percentage change in stiffness ΔR% cannot be used as an index of damage, due to the high dispersion of the data. On the contrary, there was a direct correlation between the damage index corresponding to 90% of the fatigue life and the temperature variation of the most stressed area. A ΔT equal to 34 °C is associated with a D_90%_ of 0.6, while next-to-unit damage causes a higher ΔT of 41 °C.

Another parameter taken into consideration is the heating rate during the application of the first several thousand cycles. This parameter is proportional to the amplitude of stress and is a useful parameter since it refers to the initial part of the fatigue life. It increases by an order of magnitude from a damage of 0.6 to a damage of 0.99. Future developments of this work will concern the study of different types of FRP composites in terms of composition, glass fibers and layup. Future work will include microscopic analysis to better understand the fatigue failure mechanism of composite plates.

## Figures and Tables

**Table 1:** Characteristics of the composite laminate.

% Fibers Volume	Young Modulus E_L_ [MPa]	Strain at Break ε_r_ [%]	Tensile Strength R_m_ [MPa]
57	134,000	1.41	2005
St. DEV.	2.08	0.04	66.81

**Table 2:** Fatigue test parameters.

Specimen ID	$\sigma_{\max}\;[\mathrm{MPa}]$	$\sigma_{a}\;[\mathrm{MPa}]$	Cycles to Failure	St. DEV.	Mean
**1b**	1445	650.25	9142	311,056	202,366
**2b**	1445	650.25	22,838		
**11b**	1445	650.25	663,724		
**12b**	1445	650.25	113,760		
**13b**	1360	612	5341	1,108,944	793,985
**14b**	1360	612	2,403,990		
**15**	1360	612	645,866		
**15b**	1360	612	120,743		
**7**	1275	573.75	231,627	1,714,496	1,120,118
**7b**	1275	573.75	5815		
**8b**	1275	573.75	3,667,770		
**8**	1190	535.5	2,827,231	1,889,456	2,593,927
**12**	1190	535.5	4,519,757		
**16**	1190	535.5	4,298,182		
**16b**	1190	535.5	388,787		
**17b**	1190	535.5	935,676		
**9b**	1100	495	10,000,000	0	10,000,000
**13**	1100	495	10,000,000		
**14**	1100	495	10,000,000		

**Table 3:** Percentage variation in stiffness.

Specimen	σ_max_ [MPa]	ΔR_%_	Cycles to Rupture
1b	1445	4.39	9142
2b	1445	3.41	22,838
11b	1445	8.70	663,724
12b	1445	6.07	113,760
13b	1360	4.26	5341
14b	1360	10.83	2,403,990
15	1360	9.88	645,866
15b	1360	5.80	120,743
7	1275	1.10	231,627
7b	1275	5.19	5815
8b	1275	7.00	3,667,770
8	1190	5.62	2,827,231
12	1190	10.68	4,519,757
16	1190	15.66	4,298,182
16b	1190	9.23	388,787
17b	1190	9.74	935,676

**Table 4:** Specimens analyzed by thermography and damage parameters.

Specimen	σ_max_ [MPa]	σ_a_ [MPa]	N	Heating Rate [°C/Cycle]	ΔT [°C]	ΔR_%_	D_90%_
1b	1445	650	9142	0.0110	41	4.39	0.99
2b	1445	650	22,838	0.0173	37	3.41	0.76
14b	1360	612	2,403,990	0.0098	41	10.83	0.76
13b	1360	612	5341	0.0057	40	4.26	0.88
8b	1275	574	3,667,770	0.0024	34	7.00	0.61

## Data Availability

The data presented in this study are available on request from the corresponding author.
